# Friend or foe: the dichotomous impact of T cells on neuro-de/re-generation during aging

**DOI:** 10.18632/oncotarget.12572

**Published:** 2016-10-11

**Authors:** Brandon Coder, Weikan Wang, Liefeng Wang, Zhongdao Wu, Qichuan Zhuge, Dong-Ming Su

**Affiliations:** ^1^ Institute of Molecular Medicine, University of North Texas Health Science Center, Fort Worth, TX, USA; ^2^ Zhejiang Provincial Key Laboratory of Aging and Neurological Disease Research, First Affiliated Hospital, Wenzhou Medical University, Wenzhou City, Zhejiang, P. R. China; ^3^ Department of Biotechnology, Gannan Medical University, Ganzhou, P. R. China; ^4^ Zhongshan School of Medicine, Sun Yat-sen University, Guangzhou, P. R. China

**Keywords:** T-cell immunity, aging, neurodegeneration, immunotherapy

## Abstract

The interaction between T cells and the central nervous system (CNS) in homeostasis and injury has been recognized being both pathogenic (CD4^+^ T-helper 1 - Th1, Th17 and γδT) and ameliorative (Th2 and regulatory T cells - Tregs). However, in-depth studies aimed to elucidate the precise in the aged microenvironment and the dichotomous role of Tregs have just begun and many aspects remain unclear. This is due, not only to a mutual dependency and reciprocal causation of alterations and diseases between the nervous and T cell immune systems, but also to an inconsistent aging of the two systems, which dynamically changes with CNS injury/recovery and/or aging process. Cellular immune system aging, particularly immunosenescence and T cell aging initiated by thymic involution - sources of chronic inflammation in the elderly (termed inflammaging), potentially induces an acceleration of brain aging and memory loss. In turn, aging of the brain *via* neuro-endocrine-immune network drives total body systemic aging, including that of the immune system. Therefore, immunotherapeutics including vaccination and “protective autoimmunity” provide promising means to rejuvenate neuro-inflammatory disorders and repair CNS acute injury and chronic neuro-degeneration. We review the current understanding and recent discoveries linking the aging immune system with CNS injury and neuro-degeneration. Additionally, we discuss potential recovery and rejuvenation strategies, focusing on targeting the aging T cell immune system in an effort to alleviate acute brain injury and chronic neuro-degeneration during aging, via the “thymus-inflammaging-neurodegeneration axis”.

## INTRODUCTION

Although the central nervous system (CNS) and immune systems are standalone systems, neural-immune interactions and their mutual dependency have been garnering additional attention [1, 2]. Ample evidence shows that the CNS and immune systems, particularly the T cell immune system interact, crosstalk, and affect each other across many physiological processes, including development, physiological homeostasis, disease status, and aging. The role of T cells on the CNS is generally considered pathologic, however there are many beneficial effects exerted on the nervous system [3–5]. Specifically, the well-known autoimmune demyelinating disease, multiple sclerosis (MS) [6], is a prototypical abnormal T cell immune-induced CNS pathology, however, CD4^+^FoxP3^+^ regulatory T cells (Tregs) can be exploited to exert ameliorative effects in neuro-degenerative diseases, such as cerebral ischemia [7], Alzheimer's disease (AD) [8], and Parkinson's disease (PD) [9]. The CNS itself can also, in turn, induce changes in the immune system via the neuro-endocrine-immune network [10–13]. For example, a recent report shows that activating the brain's reward system positively impacts immune responses, while ablating the sympathetic nervous system negatively impacts immune responses to bacterial load or T cell-mediated delayed-type hypersensitivity [14]. Another well-known example of CNS-induced changes in the immune system is the global immunosuppression that follows acute CNS injuries, such as cerebral ischemic stroke [15, 16]. However, the positive impact of the T cell immune system on the CNS, other than Treg control of inflammation, has been scarcely reported but is gradually becoming more recognized [17]. For example, T cells have been demonstrated to be necessary for healing brain injury [18, 19]. Even autoimmune T cells, which play a destructive role, can be beneficial to CNS functional integrity [20] and play a role of brain antigen-induced “protective autoimmunity” [21, 22]. The same subset of autoimmune T cells can be a double-edged sword to have either destructive or protective roles in neuro-homeostasis and neuro-degeneration with distinct temporal and spatial profiles [19]. For example, Tregs are generally recognized for their ability to inhibit neuro-inflammation and protect from neuro-degeneration. However, these same Tregs may also obstruct a selective gateway for immune cell trafficking to the CNS, thereby blocking neuro-recovery during acute CNS injury [23] and chronic neuro-inflammation [24]. These dichotomous affects become more pronounced in the aged microenvironment. For example, under the aging-related chronic inflammatory conditions (termed: inflammaging) healthy aged individuals have an increased susceptibility toward the development of dementia following an immune challenge [25].

The thymus is a central organ of the T cell immune system, which undergoes the natural process of aging, characterized by the progressive involution [26]. The thymus begins degenerative aging in adolescence, much earlier than other organs, and in contrast to the brain which is still in developmental stages. Therefore, the systemic age-related alterations of the T cell immune system will influence CNS homeostasis and regeneration. Although T cells are capable of circulating into the brain through the blood vessels and the recently determined CNS lymphatic vessels [27, 28], T cell entry into the CNS is rare under a normal physiological homeostasis. However, thymic involution results in not only declined output of naive T cells but also increased output of potentially harmful self(auto)-reactive T cells [29, 30]. These auto-reactive T cells have the potential to attack the selective gates protecting entry into the CNS, including both the blood-brain barrier (BBB) and the choroid plexus (CP) [31–33]. Therefore, these barriers become increasingly permeable and less selective [34, 35], progressively facilitating invasion of the CNS by a variety of T cells [35]. In addition, aging also induces chronic inflammatory conditions generated by pro-inflammatory cytokines, produced by both glial cells in the brain and senescent cells in peripheral tissues. The inflammaging condition further increases the permeabilization of CNS barriers, allowing immune cell entry into the CNS [36]. Evidence shows that immunosenescence is an early and direct trigger of brain aging and memory loss [37]. In turn, age-related neuro-degeneration in all likelihood worsens the aged T cell immune system [13, 38], perpetuating a vicious cycle of age-related multi-system degeneration. Therefore, rejuvenation of the immune system is an attractive target for therapeutics aiming to improve neuronal regeneration in the elderly.

In this article, we review recent discoveries in T cell aging that are associated with acute brain injury and chronic neuro-degeneration. We also discuss outstanding questions regarding the interplay of the aging T cell and CNS systems, and illuminate potential future studies that may help elucidate their combined roles in neuronal disease and repair. Furthermore, we identify the linked “thymic-inflammaging-neurodegeneration axis” as the prime target for potential immunotherapeutic strategies aimed to treat age-relate neuropathology.

## T CELL SUBSETS AND CONSENSUAL ROLES IN NEURAL DE/RE-GENERATION ASSOCIATED WITH AGING

**Figure 1:**
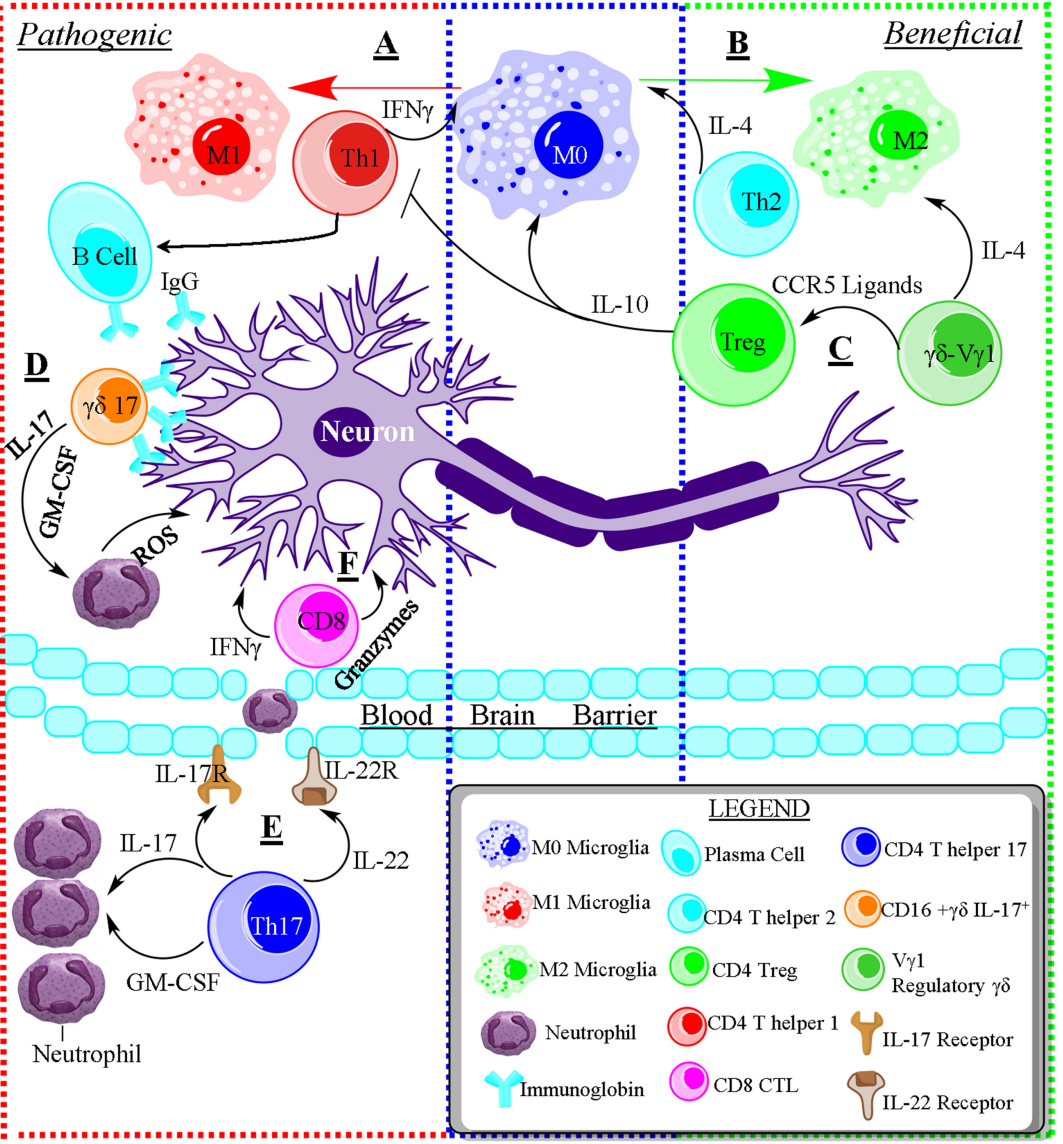
The opposing roles of immune cells in neurodegenerative disease **A**. Schematic of the pathogenic role of immune cells during neurodegenerative disease. T helper 1 (Th1) cells are capable of activating resting microglia (M0) towards the pro-inflammatory and pathogenic M1 phenotype. Th1 cells induce the production of autoantibodies from B cells, and suppress the Th2 response through IFNγ. **B**. T helper 2 (Th2) cells promote the differentiation of M0 microglia into anti-inflammatory M2 microglia that promote tissue repair. Furthermore, Th2 cells are capable of suppressing the Th1 response through IL-4. **C**. Regulatory Vg1+γδ T cells secrete IL-4 and CCR5 ligand to promote an M2 phenotype and induce the differentiation of regulatory T cells. Tregs produce IL-10, suppressing the pro-inflammatory immune response and shifting M0 towards M2. **D**. Pathogenic IL-17 producing γδ T cells can directly kill neurons through antibody-dependent cell-mediated cytotoxicity *via* binding to IgG. Additionally, pathogenic γδ T cells recruit neutrophils and induce stem cells to differentiate into neutrophils/monocytes *via* granulocyte-macrophage colony-stimulating factor (GM-CSF). **E**. Pathogenic T helper 17 (Th17) cells are also able to recruit neutrophils into the brain and induce them differentiation *via* IL-17 and GM-CSF. Additionally, Th17 cells induce the permeabilization of the blood brain barrier *via* IL-17 & IL-22 binding to IL-17R and IL-22R, respectively, on brain endothelium, allowing for the entry of inflammatory cells into the brain tissue. **F**. CD8^+^ Cytotoxic T lymphocytes are toxic to neurons by producing pro-inflammatory cytokines like IFNγ and toxic enzymes like Granzyme B.

The peripheral T cell pool is highly heterogeneous in terms of the T cell subsets, antigen specificity, and the cytokines/chemokines they produce, which are associated with functional changes in the T cell generator, the thymus, during aging. Additionally, the peripheral T cell compartment exhibits a high degree of plasticity associated with the immune microenvironment over the life of an individual, including an age-related shift towards memory and senescent CD28^null^ T cells [39], accumulation of Tregs [40], diminished T cell receptor (TCR) repertoire diversity [39], and increased frequency of autoreactive T cells in the elderly [29, 41]. Herein, we review the roles of T cell subsets on neuronal degeneration and regeneration (detrimental and beneficial affects - outlined in Figure 1), focusing on the impact of thymic involution and inflammaging on brain plasticity and maintenance.

### Characteristics of T cells produced by the aging thymus

Many of the age-related changes in peripheral T cell population dynamics are associated with thymic aging and its involution, a natural aging process, beginning in adolescence [26]. The thymus generally atrophies at a rate of 3% per year, and individuals over 50 have less than 15% of their thymic tissue remaining [42]. Thymic involution is a result of the deterioration of the thymic epithelium and results in a severe decline in naïve T cell output, which leads to decreased TCR diversity and a shift towards memory and senescent T cells [39]. In addition to ineffectiveness in response to emerging infections and vaccinations, thymic involution is also associated with increased susceptibility to autoimmune diseases as autoreactive T clones are not efficiently depleted in the involuted thymus and are instead released into the periphery. Therefore, the characteristics of the aging thymus is not only the generation of insufficient naïve T cells, but also the release of increased harmful T cells. For example, multiple sclerosis (MS), particularly patients with relapse-remission MS (RRMS), patients possess premature thymic involution with a decline in naïve T cells and increased T cell senescence [43], as well as increased autoreactive T cells.

Recently, our work reiterated that thymic involution is associated with chronic inflammation [29], which is not an overt autoimmune disease, as it lacks obvious clinical manifestations, but a condition that exacerbates the severity, incidence, and mortality of age-related diseases, including age-related neuro-degeneration. Using a mouse model of accelerated thymic involution, we found that thymic involution leads to the increased release of autoreactive T cell clones, which become activated upon encountering self-antigens in the periphery, results in cellular infiltration into non-lymphoid tissues, and leads to elevated IL-6 and tumor necrosis factor alpha (TNFα) levels.

### Dichotomous role of pro- and anti-inflamatory T cell subsets in neuro-degeneration and -protection

**Figure 2:**
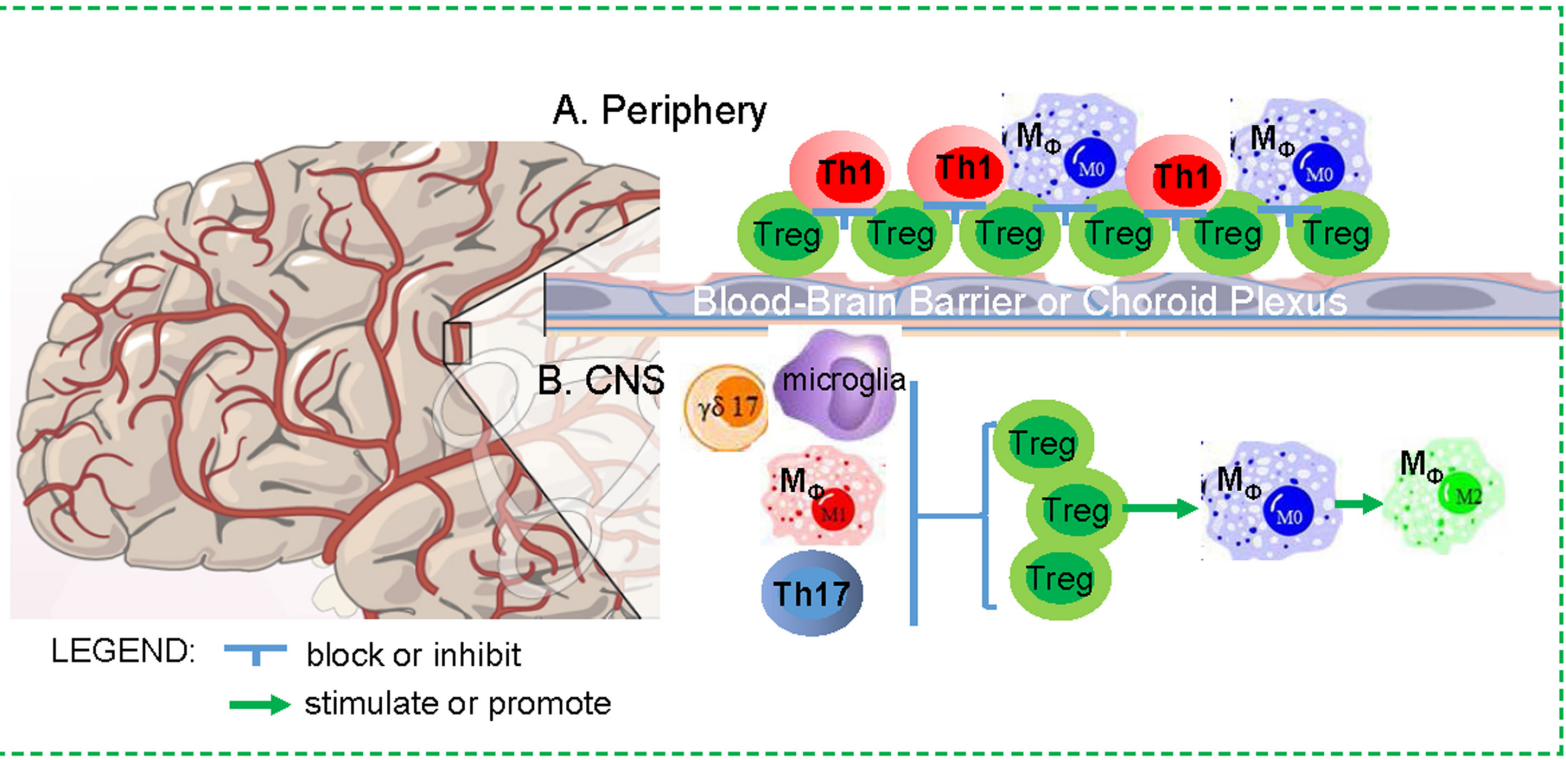
Dichotomous impacts of Treg cells on the CNS in neurodegenerative disease **A**. Periphery: Increased Tregs in the periphery in aging may result in a blockade for other immune cell trafficking through the BBB and CP, since weakening or transient depletion of Tregs is able to enhance immune cell trafficking to ameliorate neuro-inflammation in mouse models. **B**. CNS: Treg cells in the CNS may play a beneficial role, because they are able to suppress active M1 microglia and macrophage, and balance Th17 and γδ17 cells, as well as promote microglia and macrophage to M2 differentiation.

It is well known that some T cell subsets play predominately negative roles to lead to neuro-degeneration and pathology, while others exert mostly beneficial effects to facilitate neuronal protection [4, 44]. One such T-cell subset recognized as neuro-pathologic are CD4^+^ T-helper 1 (Th1) cells. Th1 cells secret Type-1 cytokines (most notably interferon (IFN)-γ and tumor necrosis factor (TNF)-α) [45], and can activate innate immune cells and CD8^+^ T cells. Th1’s, along with Th17, γδ T cells, and CD8^+^ cytotoxic T lymphocyte (CTL) cells are all predominantly involved in neurodegenerative disease and neuro-inflammation *via* pro-inflammatory cytokines [46–48] and direct cytotoxicity [49]. However, some other T cell subsets are generally considered as neuro-protective properties during neuro-degeneration, such as Th2 (producing Type-2 cytokines, such as interleukin-4, IL-4, IL-5, and IL-13) and immunosuppressive Tregs. Interestingly, recent studies have illuminated the dichotomy within these T subsets. In certain conditions, classical neuro-pathological T cells, such as Th1 cells, become beneficial and promote neuronal health, while some classical neuro-protective T cells, such as Tregs, are capable of facilitating neurodegenerative disease and neuro-inflammation. The detrimental or beneficial effects from the same T cell subset are tightly related to the localization (the CNS or periphery - See Figure 2) and CNS disease progression, and become exacerbated in aged immune and nervous microenvironments [25, 50, 51]. Th1 and Treg cells are most intrigued dichotomous subsets. Therefore, we focus on these two subsets.

### Antigen specific Th1 cells

Th1 cells have been found in the CNS of many neuro-degenerative diseases, classified as detrimental contributors to CNS pathology. For example, infiltrating Th1 cells into the CNS of MS patients are specific for self-antigen and contribute to pathology [52]. Th1 cells have been also found in the CNS of a transgenic mouse model of AD that overexpresses amyloid precursor protein and presenilin 1 (APP/PS1). These CNS-infiltrating Th1 cells are APP/PS1 antigen specific, and their production of IFN-γ was shown to lead to increased microglial activation and enhanced amyloid-beta (Aβ) plaque burden, resulting in impaired cognitive function [53].

However, Th1 cells are not always harmful in neurodegenerative diseases. In the same mouse model of AD, Aβ-specific Th1 cells were intracerebroventricularly injected into the Cerebrial spinal fluid of APP/PS1 mice, and showed to target Aβ plaques that resulted in reduction of Aβ plaque load and slight enhancement of neurogenesis [54]. This dichotomous role of Th1 cells in Alzheimer's Disease indicates that the route of migration (localization) and temporal factors may play a role in whether antigen-specific Th1 cells are beneficial or detrimental in neuro-degeneration.

Brain antigen specific Th1 CD4^+^ T cells (INFγ−producing cells) at the CP were reported to be beneficial to the brain [17] during neuro-inflammation associated with various neurodegenerative conditions including Amyotrophic Lateral Sclerosis (ALS), MS, PD, and AD. Experiments show that lacking these T cells, such as in *Rag*^−/−^ or SCID mice, impairs CNS injury repair [55] and reduces spatial learning and memory, similar to age-associated memory loss [56, 57]. The accumulation of brain-antigen-specific INFγ−producing CD4^+^ T cells at the CP is attracted by INFγ receptor, since INFγ receptor knockout mice, following spinal cord injury, exhibited reduced T cells at the CP, displayed fewer T cells entering the cerebrospinal fluid, and impaired CNS recovery [32]. The mechanism driving the accumulation of T cells into the CNS is likely involves other immune cell, such as blood-derived macrophage (M_Ø_), trafficking through the CP gate and into the CNS. The lack of IFNγ-producing Th1 cells limits the activation of the CP, thereby reducing the recruitment of M_Ø_ into the injured spinal cord parenchyma [19, 32]. Sufficient recruitment of circulating blood-derived M_Ø_ into the CNS is a key requirement for recovery from CNS injury and neuro-inflammation [33]. CNS-reactive CD4^+^ Th1 cells can be enhanced by weakening immunosuppressive Treg function in an effort to alleviate neurodegenerative disorders [58, 59]. These effects can be attributed to breaking Treg-blocked lymphocyte trafficking to the CNS through the brain CP [24]. This model of brain antigen-specific CD4^+^ Th1 cells serving as gatekeepers at the CP is very intriguing, however the mechanisms surrounding this reported phenomena are largely unclear. For example, what are the CNS-specific antigen(s)? These antigens are not likely to be the same antigens utilized in whole brain tissue or whole spinal cord homogenate antigens, which are used to induce CNS immune pathology in diseases such as Experimental Autoimmune Encephalomyelitis (EAE). Why have these auto-reactive T cell clones not been deleted in the thymus during thymocyte negative selection? What triggers the process of these auto-reactive T cells’ accumulation in the BBB or CP during CNS injury? How are these auto-reactive T cells entering the CNS without attacking the CNS parenchyma?

### Treg's dichotomous impacts on CNS functional integrity (simplified in Figure 2)

CD4^+^CD25^+^Foxp3^+^ regulatory T cells (Tregs) can develop either in the thymus (termed tTreg) or in the periphery *via* TGF-β induction (termed pTreg) [60]. Tregs primarily act to suppress effector T cells (termed Teff) in order to dampen the immune response in both auto- and foreign-source inflammation, and are capable of both antigen-specific and non-specific control of the immune response [61] and neuron-inflammation [62]. Tregs play a prominent role in slowing disease progression by decreasing pro-inflammatory cytokine levels and attenuating inflammatory M1 microglial activation *via* TGF-β−, IL-10-, and IL-4-expression [63–66]. Furthermore, Tregs can induce IL-10-producing neuro-protective M2 macrophage/microglial phenotype [66, 67]. In turn, M2 microglia can also induce antigen-specific Treg responses in EAE [68]. Therefore, many reports about Treg effects on attenuation of neuro-inflammation are positive. For example, levels of FoxP3 expression, Treg numbers and Treg function are impaired in patients with rapidly progressing ALS [69] and relapse-remission MS patients [70, 71].

However, Tregs have also shown to play a detrimental role in the CNS during acute CNS damage [23] and chronic neuro-inflammation [24]. Recent reports show that transient depletion of FoxP3^+^ Tregs displayed amyloid-beta (Aβ) plaque clearance, neuro-inflammation amelioration, and cognitive decline in AD mouse model [24]. Additionally, increased FoxP3 expression correlates with *Tau* protein levels in the CSF of AD patients [72]. Furthermore, Treg frequency is elevated in the elderly [72], but this is no help for controlling age-related neurodegenerative diseases. Therefore, weakening Treg function to break self-tolerance to CNS antigens has been a proposed strategy for fighting chronic neuro-inflammatory disorders [24, 73].

The dual natures of Tregs on CNS diseases are probably due to the existence of distinct Treg subsets or Treg clones. For example, a decrease of PD1^-neg^ Tregs, which is considered as functional Treg subset with the highest suppressive capacity, was seen in patients with severe AD compared to those with mild cognitive impairment, implicating a distinct highly suppressive Treg subset in controlling AD severity [74]. Another example, FoxA1^+^Tregs (a novel Treg subset with transcription factor FoxA1 ectopic expression) can efficiently suppress EAE in both FoxA1-dependent and programmed cell death ligand (PD-L)1-dependent manners [75]. Emerging evidence reveals clonality of Treg and the distinct sets of antigens they recognize. The absence of Tregs in certain tissues of autoimmunity regulator (*AIRE*) gene deficient animals indicates that the generation of a subset of Tregs is *AIRE* gene dependent [76]; while other works have shown the generation of *AIRE*-independent Tregs in *AIRE* knockout animals [77, 78]. Because AIRE controls the expression of a particular set of promiscuous self-antigens in the thymus, *AIRE*-dependent and *AIRE*-independent Tregs are likely to recognize a completely distinct TCR repertoire of self-antigens. Interestingly, the majority Tregs emigrating from the aged thymus are likely to be *AIRE*-independent, since Treg generation in aged thymus is not impaired, however, *AIRE* gene expression is decreased [29, 41]. Whether these aged Treg clones preferentially skew toward a particular set of self-antigens (e.g. CNS derived antigens) needs further investigation. Another potential cause for Treg's dichotomy in relation to the CNS is probably due to impaired migration into the CNS in neurodegenerative diseases. While Tregs can be detected in the peripheral blood of MS patients, their absolute numbers in MS brain lesions were extremely low or undetectable, suggesting that Treg migration into the brain is impaired or Tregs undergo apoptosis in MS lesions [79]. Treg migratory capability in RRMS patients was also significantly impaired [80]. The third possibility of Treg's dichotomous impacts on the CNS could be Treg's localization - outside or inside of the BBB (Figure 2). If they are located outside the BBB, they block other immune cell trafficking, whereas if they enter into the CNS, they play a role to inhibit inflammatory cells. Overall, exploration of the dichotomous role of Tregs on CNS functional integrity is in its infancy and includes many outstanding gaps in knowledge that must be further investigated.

## T CELL RESPONSES FOLLOWING ACUTE CNS INJURY, AND AGE-RELATED HOMEOSTASIS RELATED TO CHRONIC NEURO-DEGENERATION

Roles of individual T cell subsets involved in the integrated T cell immune responses in different neuronal diseases, such as CNS injuries and chronic neurodegenerative diseases, are different. Their roles become more intricate in aged microenvironment. Except for acute cerebral trauma from an external blow to the head, most brain injuries and neurodegenerative diseases are directly and/or indirectly associated with age. For example, acute brain ischemic stroke is usually induced by insufficient blood flow to the brain likely owing to age-related blood vessel and flow abnormalities (indirect factors), while chronic neuro-degeneration is mostly associated with chronic inflammation during the aging process (direct factors). Furthermore, although autoimmune demyelinating disease MS is not associated with aging (onset at 20 - 40 year olds), it tends to worsen with aging (over 40 year olds). And MS patients frequently display accelerated aging of the thymus. Therefore, how inflammaging develops and its direct/indirect role in acute and chronic neural diseases will be discussed.

### Immunopathological characteristics in acute and chronic neural diseases associated with aging

T cell-mediated immune responses or immunopathology are involved in virtually all types of age-related CNS disorders, including acute brain injury induced by blood flow abnormalities; autoimmune demyelination MS induced by autoreactive T cells; unknown etiology neuro-degeneration ALS, and chronic neuro-degeneration AD and PD caused by accumulated misfolded protein-induced neuro-inflammation associated with inflammaging. However, these T cells exhibit distinct immunopathological characteristics within the different neuronal disorders, and their accumulation can either be considered part of an etiology and/or as an outcome of neuronal disease

In post-cerebral ischemic stroke, infarct brain tissue triggers an immunopathogenic inflammatory cascade, involving both the innate and adaptive immune responses. The responses display a complex interplay between the CNS and the immune system, and lead to both amplification of local inflammation in the brain and secondary cerebral damage [81–83], characterized by neuro-degeneration [7]. Although innate immunity-induced inflammation by the resting microglia and blood-derived M_Ø_ cells is well accepted, the dynamic T cell responses after stroke are beginning to garner more attention [7, 82]. Two major T cell-related immune mechanisms are involved in post-ischemic stroke-induced inflammation. First one, the CD4^+^ Th1 cell-dependent pro-inflammatory pathway (see section 2.2a), in which IFNγ mediates polarized immune response leading to inflammation and exacerbates brain injury. Second mechanism, the interleukin-17 (IL-17)-induced inflammation has received increasing attention in recent years. IL-17 is predominantly produced by γδ T cells in this inflammatory process [84]. Neutralization of the IL-17 axis has been reported to be able to diminish brain damage [85]. Both γδ IL-17 and Th1 pro-inflammatory pathways synergistically enhance recruitment of neutrophils into the CNS to lead to secondary cerebral damage [85]. Additionally, CD8^+^ T cells, which are recruited as early as 3 hours after stroke onset, and NK cells (recruited within 24 hours) are all involved the acute inflammation [86]. On the opposite spectrum, anti-inflammatory Th2 cells [87–90] and Tregs [91] are generally regarded to be able to protect from post-ischemic stroke inflammation-caused brain injury.

Multiple sclerosis (MS), a self-reactive CD4 T cell-mediated demyelinated autoimmune inflammatory CNS disorder, is classified as a prototypic heterogeneous autoimmune condition, mainly mediated by autoreactive Th1 cells and pathogenic Th17 cells [44, 52]. Although symptoms emerge in young adults between the ages of 20 - 40 years old, patients with MS undergo an age-related acceleration in progressive axonal loss, potentially due to the synergistic effects of age and neurologic illness [92, 93]. The pathology of MS is typically associated with premature thymic involution in RRMS [94, 95]. The role of Tregs in disease progression, either deleterious or protective, remains uncertain. Tregs are usually considered to play a protective role [96] to balance Th17 in MS [97, 98], and this balance is broken with Treg reduction in MS. However, using humanized monoclonal antibody Daclizumb, which is an anti-CD25 antibody with a potential to block Tregs (reduction of 60% of Tregs in a 4-day dosage [99]), to treat MS patients has received positive clinical effects [100, 101], implying that Tregs may facilitate MS progression.

Similar to MS in many aspects, Amyotrophic lateral sclerosis (ALS), is an age-related neurodegenerative disorder that is no considered an autoimmune disease, however the associated neuro-inflammation stemming from the interplay between microglia and infiltrating T cells is thought to play a major role in pathogenesis [102]. ALS is typically correlated with the onset of prior autoimmune disease [103], and both T cells and humoral antibodies were found to affect the motor-neurons [44, 104]. Additionally, Tregs were observed to increase with ALS disease onset, but in the later stages of disease, the number and suppressive function of these Tregs were reduced [65, 66]. However, not all antigen specific T cell responses are associated with ALS progression. Using the “protective autoimmunity” strategy [21] to immunize mSOD1 mice with a myelin-derived antigen has been shown to attenuate ALS disease progression. There appears to be an indirect mechanism facilitating “protective autoimmunity”, associated with opening the brain's choroid plexus (CP) gate to recruit peripheral Tregs and IL-10-produing M_Ø_ cells [105].

Age-associated chronic neurodegenerative diseases AD is the leading cause of dementia [106, 107], however, its etiology is unknown. The pathological features of AD have been well characterized, including extracellular deposition of amyloid-beta (Aβ) protein and intracellular accumulation of neurofibrillary tangles generated by abnormal hyper-phosphorylated Tau protein [108]. Although T cells are found in the brain of AD patients [106, 109], and have been implicated in the induction of AD or enhancement of the disease, their overall function remains unclear [110]. Mounting evidence indicates that misfolded proteins Aβ and Tau can stimulate the immune system to activate resident immune cells such as microglia and astrocytes [111, 112], and to increase retinoic acid-related orphan receptor (ROR)γt^+^ T cells (Th17) and nuclear factor of activated T (NFAT)c1^+^ CD4 cells [46], that in turn release inflammatory mediators, ultimately exacerbating AD progression [113, 114]. The adoptive transplantation animal model (APP/PS1 mice) has helped to elucidate the role of three subsets of T cells in AD [8]. Transplantation of Th1 cells exacerbated the disease [53], while transplantation of Aβ-specific Th2 [115] or xenogenous Treg cells [116] ameliorated the disease, including improved cognition, reduced plaque deposition, and decreased Aβ burden. However, the mechanism of Treg protection and suppression of neuro-inflammation remains elusive, largely because the targeted effector cells are unknown. Additionally, it remains unclear why the age-related accumulation of highly suppressive Tregs [72] are not able to attenuate neuro-inflammition in the elderly, and in many cases appear to exacerbate disease. Therefore, the dichotomous role of Tregs in age-related neuro-degenerative AD needs to be further assessed.

Like AD, Parkinson's Disease (PD) is another age-related neurodegenerative disease [117], with the progressive clinical motor symptoms, which are considered to be the result of loss of dopamine neurons in the substantia nigra pars compacta. The pathological hallmark of PD is intracellular deposition of Lewy bodies and Lewy neurites, which contain a fibrillar and misfolded protein called α-Synuclein (α-Syn) [118], responsible for inducing a complex immunopathogenic response. Although the etiology of PD is unknown, onset and progression of the disease result from the interplay between the innate and adaptive immune systems, evidenced by a significant reduction in dopaminergic neuron death in immunodeficient mice [119]. Knock-out of major histocompatibility complex (MHC) Class-II (MHC-II) has been shown to prevent α-Syn-induced microglia activation, antigen presentation, IgG deposition, and the degeneration of dopaminergic neurons [120, 121]. Many T cell subsets have been observed in PD pathogenesis: adoptive transfer of Th1 and Th17 cells derived from α-syn-immunized mice leads to exacerbation, while transfer of Tregs leads to attenuation of neuro-degeneration in mouse model of PD [122].

### Inflammaging and pro-inflammatory factor production

Virtually all age-related diseases, either arise from or, are exacerbated by “inflammaging”, which is a low-grade, but above base-line, and sustained chronic inflammation associated with aging [123–127]. Although the etiology is not fully understood, inflammaging has been attributed to a combination of cellular senescence-induced “senescence-associated secretory phenotype (SASP)” (details as below) that releases low levels of pro-inflammatory cytokines, such as IL-6, TNFα, IL-1, and C-reactive protein (CRP), [126, 128–130], and the persistent activation of immune cells by chronic viral infections like cytomegalovirus (CMV) - so-called “foreign-reactive” immune cells [125, 127, 131–133]. Additionally, we found that auto-reactive T cells contribute to the emergence of an inflammatory state with advanced age, which is associated with tissue damage inflicted by so-called “self-reactive immune cell-induced damage of self-structure tissues”. We showed that thymic involution, a natural feature of the aging process, is on its own sufficient to induce chronic inflammation. In a conditional knockout of *FoxN1* to induce thymic involution mouse model, recently emigrated T cells from the atrophied thymus were capable of reacting to self-antigens and becoming activated in the periphery, which ultimately led to inflammatory infiltrates in non-lymphoid organs and increased production of the pro-inflammatory cytokine TNF and increased levels of serum IL-6 [29, 41].

### Senescence-Associated Secretory Phenotype (SASP)

SASP [134] likely contributes to tissue degeneration, including neuro-degeneration. Although the exact characteristics of senescent cell types in the aging brain are unclear, mounting evidence indicates that astrocytes and microglial cells potentially grow senescent with advancing age [135]. Astrocytes from aged rat brains stain positive for senescence-associated beta-galactosidase (SA-βGal) and have increased expression of the senescence molecules p21 and p16^INK4a^ [136]. Microglia undergo telomere shortening with advancing age [137], which can lead to cellular senescence. Nevertheless, SASP pro-inflammatory factors create a persistent low-level inflammatory environment with advancing age that can profoundly affect neighboring cells and systemic milieus, and induce and/or enhance neurodegenerative diseases such as AD [138] and PD [139]. Although the precise molecular mechanism of the SASP signaling pathway is unknown, activation of the DNA damage response (DDR), p38 mitogen-activated protein kinase (p38MAPK), and mechanistic target of rapamycin (mTOR) to trigger nuclear factor kappa-light-chain-enhancer of activated B cells (NF-κB) and CCAAT/enhancer binding protein transcription factors are the likely contributors [126, 134, 140]. Importantly, activation of NF-kB signaling, a hallmar of immune cell activation, is a major trigger of SASP in senescent cells [141, 142].

### Macrophage (M_Ø_)/microglia and inflammasomes

In brain trauma and ischemia-elicited brain acute inflammation, the cerebral resident microglia and recruited blood-derived M_Ø_ [143] play a major role in both cleanup of tissue debris and modification of inflammation. In response to environmental stimulation, microglia and M_Ø_ are activated to differentiate into two types - M1 and M2 [144, 145] with physiological and functional differences. M1 cells clear debris and induce inflammation [146], whereby the inflammasomes, typically, nod-like receptor protein (NLRP)3 and NLRP1, are activated to trigger increased production of pro-inflammatory factors such as IL-1β and IL-18 [147], and nitric oxide synthase (NOS2) [148]. In the chronic neuro-inflammatory aging environment, the inflammasome-mediated inflammatory pathway also plays an instrumental role in causing and/or aggravating neuro-inflammation [149]. For example, NLRP3 polymorphisms were found to be associated with late-onset AD [150].

Although NLRP3 is activated in the microglia and M_Ø_ in the brain, it is also potentially activated by senescent cell secreted pro-inflammatory cytokines. It is proposed that upregulated NF-kB signaling, which is a major inducer of SASP, during aging could potentially initiate the NLRP3 inflammasome in the brain [151]. Therefore, NLRP3 activation-induced increase in IL-1β levels can be seen not only in acute infection, brain trauma, and ischemia, but also in the aged brain with chronic inflammation, such as what has been observed in AD [152]. Recently, a new stress-induced intra-neuronal inflammasome activation pathway (NLRP1/Casp1/Casp6) was reported in AD patients, in which NLRP1 activation triggers Casp1 activation to induce IL-1β maturation, while Casp1 activation also induces Casp6 activation to mediate axonal degeneration. It is evidence that in the AD brain, NLRP1^+^ neurons were 25- to 30-fold higher than in non-AD brains [153].

Opposing the inflammatory and inflammasome driven M1 effector functions, the M2 phenotype is involved in anti-inflammation *via* immune modulation [154–156]. If NLRP3 becomes inactivated, the microglia and M_Ø_ display M2 phenotype with increased IL-4 and Arg1, and decreased pro-inflammatory production [152]. Both M1 and M2 types of microglia and M_Ø_ are required for recovery from CNS injury [157]. Furthermore, these classifications are not completely binary, as there is a switch from an M1- to M2-dominant response, involving cleanup of brain cellular debris in the early stage and anti-inflammation at the later stage of recovery [156].

## POTENTIAL IMMUNOLOGICAL STRATEGIES FOR REJUVENATION AND CURE OF AGE-RELATED NEURO-DEGENERATION

Although effective immunotherapeutic options, particularly treatments for acute CNS injury and chronic neuro-inflammation, remain limited, interest in this field is rapidly increasing. Development of immunological therapeutic strategies for the best rejuvenation of age-related neuro-degeneration should target risk/inflammatory factors and focus on etiologies. Using anti-inflammatory drugs to modulate innate and adaptive immune reactions is a known approach. For example, long-term use of anti-inflammatory drugs reduces the risk for AD and PD by roughly half [158–160], whereas alteration of peripheral inflammation during neurodegenerative disease can significantly alter the disease course [161]. However, the immune components, including various types of T cell subsets, are garnering more attention, since they have been observed in both the induction and suppression of chronic inflammation. Therefore, attenuating neuro-inflammation is tightly dependent on rebalancing these activated immune cell populations.

### Improvement of cellular microenvironment-based therapy

Improvement of the neuron stem cell (NSC) and immune cell microenvironment is a therapeutic strategy that has the potential to recover acute CNS injury and rejuvenate homeostasis of chronic neuro-inflammation in the elderly. Most recently, there are two pivotal progresses in this field.

#### Shifting an M1- to an M2-dominant response

As we discussed in previous section, both M1 and M2 types of M_Ø_ and microglial are required for acute CNS injury and chronic inflammatory neuro-degeneration [157]. During the early stages of the recovery process blood-derived M_Ø_ enter the CNS, which cannot be replaced by the CNS resident microglia [143]. Although M1 cells have pro-inflammatory activity and are largely detrimental to the CNS, this is necessary for cleaning up CNS cellular debris. At later stages of the recovery, M2 cells are dominate the M_Ø_ landscape. M2 cells have anti-inflammatory effects and are beneficial in reducing secondary damage to the CNS [162], thereby the switch from an M1- to M2-dominant response halts the inflammatory process [156]. Aging induces cellular senescence, and M_Ø_ undergo senescence similar to other cell types. Unlike young blood-derived M_Ø_, which efficiently rejuvenate regeneration in the aged injured CNS through “heterochronic parabiosis” (surgically joined young and old mice) [156, 163], aged M_Ø_ are less efficient at cleaning up cellular debris during CNS injury recovery [163] and much slower at switching from an M1- to M2-dominant response [156]. Studies are attempting to reduce the time taken for the transition from M1- to M2-dominant response, in order to shorten the pro-inflammatory process and prolong anti-inflammatory effects. For example, intracerebroventricular infusion of rapamycin, an inhibitor of mTOR signaling, enhanced brain M_Ø_ polarization to M2-dominant response, and reduced γδ T cell and granulocyte infiltration into the CNS to attenuate CNS secondary damage after ischemic stroke [164]. mTOR is a kinase linking growth and aging [165], and T cell development and activation [166] through myriad signals. Inhibition of mTOR signaling was also reported to inhibit differentiation of Th17 cells and promote the generation and activation of FoxP3^+^ Treg cells, which are beneficial to CNS injury recovery [167, 168]. Another example is the administration of IL-4 to enhance the M2-dominant response in the CNS after intracerebral hemorrhage [169]. Evidence shows that aged mice are less sensitive to the M2-promoting effects of IL-4 in Lipopolysaccharides (LPS)-induced neuro-inflammation [170]. Use of IL-4 in aged individuals during CNS injury as a means of treatment may be potentially helpful, which is reviewed elsewhere [4], but dosage may present a hurdle.

#### Tregs and Treg-derived exosomes for therapeutics

The evidence of Treg-based therapy in recovery from acute CNS injury, such as cerebral ischemic stroke, was established using two different approaches. First, Treg depletion was shown to impair recovery in post-ischemic stroke by augmenting the activation of CNS resident and invading inflammatory microglia/M_Ø_ and T effector cells, and elevation of pro-inflammatory cytokines (TNFα, IFNγ, and IL-1β) in the brain [171]. However, these phenotypes were not reproducible using a different Treg depletion method by another group [172]. This is mainly attributed to complete [172] or partial [171] depletion of Tregs, where incomplete depletion may be more harmful for recovery from post-ischemic stroke-associated lesions [171]. Second, many experiments have indicated that enhancing Tregs could reduce brain damage in post-ischemic stroke. This can be achieved through several approaches: (1) adoptive transfer of Tregs into ischemic stroke individuals [173]; (2) amplification of the host's own Tregs by intraperitoneal injection of a CD28 super-agonistic monoclonal antibody (CD28SA) [174]. The CD28SA can efficiently expand and activate polyclonal Tregs *in vitro* and *in vivo* without TCR engagement [175, 176]; (3) boosting the host's own Treg suppressive function by enhancing IL-10 expression through injection of trichostatin A [177]. Trichostatin A is a histone deacetylase inhibitor, which can epigenetically activate the FoxP3 gene and promote the generation and function of Tregs [178]; (4) transplantation of bone marrow mesenchymal stem cells (BMSCs) or neuron stem cells into MCAO animals induces an increase in cerebral Tregs [179, 180], although the mechanism is unclear. In addition to their therapeutic role in acute CNS injury, Tregs have been shown to affect the attenuation of chronic inflammation-induced neuro-degeneration [72, 122]. Furthermore, vaccination can facilitate Treg therapy of autoimmunity-induced neuro-degeneration, such as in EAE [181]. The myelin oligodendrocyte glycoprotein peptide_35-55_ (MOG_35-55_) can be used as a self-antigen for use in a DNA-vaccine to induce Tregs and anti-self-antigen-specific immune responses [181, 182]. This therapy was demonstrated to be safe and effective, and is being tested in a clinical trial for MS [183, 184]. Clearly, the therapeutic use of Tregs in CNS injury and autoimmune disorders is an area of growing interest [185, 186].

However, a recent report using naïve rats described a high proportion of FoxP3^+^ Tregs in the CNS that are able to suppress Lipopolysaccharides (LPS)-induced inflammatory responses of brain microglia/macrophages [64]. The question arises as to why additional Tregs are required for the therapy of CNS injury and chronic inflammation-induced neuro-degeneration, when the CNS already has a high proportion of resident Tregs? Is this because the cerebral Tregs are reduced during acute and chronic CNS damage? Except for MS, in which Tregs are impaired [187], neurodegenerative diseases, such as AD and PD, are reported to have increased Tregs with higher activation in the peripheral blood [72], that likely suppress T effectors of pathological proteins such as Aβ and tau proteins in AD. Therefore, an excessive increase in Treg number could potentially perturb the balance of beneficial immune responses necessary cleaning up accumulated pathological proteins and debris. Therefore, it will be of great interest to re-evaluate whether enhancement of Treg number and activation in acute and chronic CNS inflammation are really necessary for their therapeutic use.

In addition, Treg-based therapy is riddled with other unsolved issues. For example, if Tregs from an individual other than the host are used, then there is an MHC matching requirement between the two individuals, making the expansion of autologous Tregs likely to be necessary for cell-based therapy. In the case of expansion of Tregs in the elderly, there is the issue of senescent Tregs and altered function. The immune periphery of aged animals and humans have an accumulation of Tregs [40], resulting from a decrease in pro-apoptotic *Bim* gene expression [188–190]. Although aged Tregs were not found to be impaired in their suppressive function [191], progressive aging does affect some Treg subpopulations [192–194]. Whether aged Tregs are the equivalent to young Tregs at the individual cell level or whether they are associated with replicative senescence is unknown [195, 196]. Therefore, cell-free transplantation through Treg-derived exosomes may be a better strategy in Treg-based therapy [197–199].

Exosomes are small membrane vesicles of multivesicular bodies secreted by numerous cell types, including FoxP3^+^CD4^+^ Tregs [200]. Exosomes bear soluble epigenetic components, including mRNAs, microRNAs (miRNAs), other noncoding RNAs, lipids, and proteins from their originating cells, of which miRNAs and other small RNAs are the most abundant components [201, 202]. Exosomes are epigenetic regulators that induce or suppress gene expression *via* intercellular communication. Each cell type-derived exosome contains a distinct pool of components with distinct functions. Treg-derived exosomes are distinct from those of Th1 and Th2 cells, and necessary for controlling systemic inflammation via suppression of pathogenic Th1 cell proliferation and inhibition of IFNγ production [197]. Notably, Treg-mediated exosomal delivery of miRNAs and other immunoregulatory factors should have suppressive and immune modulatory functions similar to that of cell-contact mediated Tregs, and are potentially therapeutic [199] in CNS injury and inflammatory recovery.

#### Immunization (vaccination) therapy

Conventional vaccination prevents infectious diseases by the administration of antigenic material (a vaccine) to develop an individual's adaptive immunity (mostly antibodies) against specific pathogens. Nevertheless, vaccination is also used for treatment (cure instead of prevention, termed therapeutic vaccination) of neuro-degeneration, neuro-inflammation, and tumors, which are not infectious diseases and involve the establishment of not only antibodies, but also cellular immunity. With regard to neuronal diseases, therapeutic vaccination is not only applied in autoimmune neuro-degeneration, such as MS, but also in inflammatory neuro-degeneration, such as AD and PD, and in traumatic CNS injury.

#### Vaccination against autoimmune T cells in MS

In MS, the CNS is attacked by abnormal autoimmunity through an inflammatory demyelinating disorder, in which myelin-reactive T cells are involved. Therapeutic vaccination, including the use of T cell vaccine (TCV), TCR peptide vaccine, myelin basic protein-based DNA vaccine, and altered peptide ligand vaccine, shows great promise in ameliorating the disease [203], not only in rodents (A rodent model for human MS is EAE.), but also in humans [204, 205]. The most promising approach uses attenuated autologous myelin-reactive TCV, with attenuated autoreactive T cells from MS patients to induce T cell-dependent inhibition/neutralization of disease-causing T cells, and regulation of the autoimmune response [206, 207]. The T cells for TCV therapy are usually derived from the patient, attenuated with irradiation, and then re-injected into the patient to elicit an immune reaction or immune regulation, in order to reduce or eliminate myelin-reactive effector T cells, decrease Th1 cytokine (such as INF-γ)-producing cells in the CNS [208], and increase and activate CD4+ Tregs, which can inhibit autologous myelin-reactive T cells [209, 210], thereby ameliorating inflammation and disease.

In addition, immunization with a myelin-derived antigen was reported to be able to activate the brain's choroid plexus (CP), thereby enhancing recruitment of immunoregulatory cells to the CNS to achieve attenuating ALS disease progression in a mouse model [105]. The target of this immunization is not disease-related T cells, but the patient's CP. The vaccine was shown to induce the CP to express IFNγ and attract Th1 cells, which is demonstrated to be required for CNS immune surveillance and repair [32]. However, further in-depth studies and additional molecular evidence for this mechanism are required.

#### Therapeutic vaccination, Aβ- and Tau-based immunotherapy

Age-related chronic neurodegenerative diseases such as AD and PD are characterized by the accumulation of pathogenic misfolded proteins. Therefore, immunization to target epitopes from these proteins is a potential effective strategy. Therapeutic vaccination for AD is to target two abnormal protein: extracellular deposition of misfolded Aβ and Tau proteins [211, 212] to produce specific antibodies for enhancement of clearance by inducing phagocytosis [213] and neutralize the toxic effects [214], and inhibition of anti-oligodendrocyte- and myelin-related neurite outgrowth [215].

There are two major ways to elicit these antibodies. One is active immunity with Aβ or Tau peptides (such as using immunoge Aβ_1-40/42_) to elicit antibodies from patients themselves; the other is passive immunity by transfer of Aβ− or Tau-specific antibodies made from others, e.g., humanized monoclonal antibodies, to patients.

Therapeutic vaccination takes advantage of the immune response against a harmful self-antigen, but there is a risk of causing adverse autoimmunity. For example, active immunization with Aβ_1-42_ peptide for AD immunotherapy induced the onset of meningoencephalitis in 6% of treated patients [216]. The underlying mechanism is thought to be due to T cell-mediated autoimmunity, which is attributed to the Th1-biased adjuvant, QS-21 [212, 216]. Therefore, the passive immunotherapy using humanized monoclonal antibody (mAb) is thought to be relatively safe [212, 217], and several mAbs have been tested in clinical trials on humans [218]. More and more groups are seeking safe and effective immunogens for vaccination to minimize autoimmune reactions and switch immune response type in developing a cure for AD, such as using non-viral DNA vaccines without any adjuvant [219] to rein in excessively strong immune reactions and to elicit a Th2-type immune response in the host [220, 221].

Unlike Aβ in AD brain that forms extracellular senile plaques, aggregated hyper-phosphorylated Tau protein forms intracellular neurofibrillary tangles. In recent years vaccines designed to clear aggregated hyper-phosphorylated Tau protein are being developed for the immunotherapy of AD [222–224], exhibiting effective responses against Tau in animal models of AD [225–227]. Additionally, passive immunization to target Tau using specific Ab in mouse models has been demonstrated [228, 229]. However, since Aβ and Tau induce different pathological changes in the brain of AD patients, whether simultaneously targeting both proteins will produce a synergistic therapeutic effect has just been tested with promising effects [230]. However, more reports in this field are required for establishment.

The development of a vaccine for PD employs a similar concept as for AD, with great progress in recent years [231]. PD pathology in the brain is characterized by Lewy bodies, in which the major constituent is the accumulated and aggregated misfolded synaptic protein - α-synuclein (α−Syn). Therefore, cerebral α−Syn protein was selected as the target [232]. Active immunization with a full length α−Syn-based vaccine [233] or passive immunization with an α−Syn-specific monoclonal antibody [234, 235] were tested in a mouse model over-expressing human α−Syn protein, with both approaches showing efficacy in disaggregating transgenic human α−Syn protein. Promisingly, active immunization for human PD patients has been developed [236]. To avoid autoimmune reactions, such as antibody-induced meningoencephalitis in AD immunotherapy [216, 237], this PD vaccine uses a short peptide (7 amino acids) in order not to induce an α−Syn-specific T cell response, but provides the T helper epitopes in carrier proteins to sufficiently activate the B-cell response.

Therapeutic vaccination also holds promise in other CNS degenerative diseases [238], such as acute traumatic brain injury [239] or chronic psychiatric conditions [56]. The use of vaccination to cure CNS injury is based on the principle of immune suppression of harmful proteins. When the CNS is acutely injured by either trauma or stroke, myelin and oligodendrocyte associated neurite growth inhibitors, such as Nogo-A [240] and myelin-associated glycoprotein (MAG) [241], are released into the CNS environment and inhibit neuron, particularly axon, regeneration. Antibodies specific to these inhibitors can reverse and promote neurite outgrowth [242, 243]. Tau protein is also phosphorylated following traumatic brain injury, and potentially leads to a pathological form of tauopathy-related dementia [239]. Therefore, Tau vaccination holds great promise as a therapeutic strategy for traumatic brain injury.

#### Harnessing “autoimmune therapy” for neuro-regeneration and CNS injury recovery

Dr. Schwartz's group, with decades of experience on the cross-talk between the immune and nervous systems, found that therapeutic vaccination with self-antigens, such as MBP, can play a role in neuro-protection and promote repair, renewal, and rebalance of neurodegenerative conditions. They termed this mechanism “protective autoimmunity” or “T cell immunity to self-maintain the self” [21, 244]. While autoimmunity is harmful to a healthy body, the key to harnessing it for therapy lies in controlling the antigen, carrier, timing, dose, and regimen. For example, using part of MBP peptide-51 - 70 as an antigen is safer (does not induce EAE) and more effective (does not disrupt retinal ganglion cells) than using whole MBP [21]. The “protective autoimmunity” confers benefits not only in therapy of CNS trauma, such as spinal cord injury [51, 245] and cerebral ischemic stroke [246], but also in spatial learning and memory [57], since mice deficient in total or CNS-specific T cells exhibited reduced spatial learning and memory capabilities, which were restored by providing them with WT T cells [56, 247]. Two approaches are currently used in therapeutic vaccination with self-antigens — injection of antigen, such as spinal cord homogenate (SCH), directly into the host animal [51], or injection of dendritic cells, which are primed by SCH, into the host animal [248]. The precise mechanism, by which self-antigen induces “protective autoimmunity”, is still unclear, but two plausible theories may explain the mechanism: (1) to remove toxic materials generated during CNS injury, such as rapid induction of autoantibodies against Nogo-A, the encephalitogenic MOG, and neurite growth inhibitors [249]; (2) to recruit blood-derived M_Ø_ cells to the CP [55] and facilitating their entry into the CNS through the BBB [31, 33], which is compromised during CNS injury. The M_Ø_ is necessary to clean up the damaged cellular debris (M1 function) [163] and execute immune modulation (M2 function) [162] in the injured CNS. However, there remains a pressing need in “autoimmune therapy” to balance and augment the beneficial response without eliciting detrimental autoimmune pathology and aggravating inflammation. As the field of “protective autoimmunity” progresses, there will likely be a need to design kill-switches into the therapy in order to shutdown off-target autoimmune reactions that may arise.

## CONCLUDING REMARKS

Our understanding of neural-immune crosstalk and mutual influence in aging and age-related diseases has advanced greatly over the past decade. This review of immunology and neurobiology in the context of aging and age-related diseases aims to stimulate the development of new therapeutic strategies. We focus on the impact of cellular immune system aging on neurodegenerative diseases, and the great promise that immunotherapy holds for the rejuvenation and cure of these diseases with the goal of developing novel and practical therapeutic strategies in elderly individuals at risk for chronic inflammation-associated cardiovascular and neurodegenerative diseases. Although enormous progress has been made, many challenges and questions remain, and further investigation is critically needed. Further investigation should focus on (1) The etiology of inflammaging-associated chronic neuro-inflammation, which remains largely unclear. Although all aged persons exhibit some levels of chronic inflammatory conditions, not all individuals suffer from age-related diseases, such as AD or PD. This is because inflammaging is necessary but not sufficient to induce age-related neurodegenerative diseases. Clearly, an additional trigger is required for disease onset, and the nature of the trigger(s), be it infection, nutrition, or other environmental factors, needs to be investigated. (2) Fundamental mechanisms by which a detrimental immune reaction takes place in the CNS associated with inflammaging or secondary cerebral damage during acute CNS injury are still unclear. The lack of a T cell immune reaction has been confirmed to impair CNS injury recovery and disrupt maintenance of brain plasticity. Therefore, the immune cell types (such as Th1/2/17 types and M1/M2 types), their self-reactivity, and their migration into the appropriate location (such as at the brain parenchyma or CP) and timing (such as length of M1/M2 response transition) will probably be tightly associated with disease onset. (3) Although immune interventions hold great promise for the treatment of neurodegenerative diseases, the efficiency of these immune therapeutic strategies needs to be improved since developed vaccines do not yet cure or efficiently treat AD and PD. One aspect that has been overlooked is that vaccination of immunosenescent patients is not efficient and all elderly patients exhibit some degree of immunosenescence [39]. Therefore, vaccination and rejuvenation should be considered in concert. We have great expectations that in the near future, progress in the understanding the etiology and mechanisms of age-related neuronal disorders will lead to novel and efficient immunotherapeutic strategies.

## Abbreviations

Aβ: amyloid-beta
AD: Alzheimer's disease
ALS: Amyotrophic Lateral Sclerosis
APP/PS1: amyloid precursor protein and presenilin 1
α-Syn: α-synuclein protein
BBB: blood-brain barrier
CNS: central nervous system
CP: choroid plexus
CSF: cerebrospinal fluid
CTL: Cytotoxic T Lymphocytes
EAE: Experimental Autoimmune Encephalomyelitis
Fas: FAS receptor: apoptosis antigen 1
FasL: FAS ligand
HIV: human immunodeficiency virus
IFN: interferon
IL-: interleukin-
MØ: macrophage
MBP: myelin basic protein
MCAO: middle cerebral artery occlusion
MHC: major histocompatibility complex
MOG: myelin oligodendrocyte glycoprotein
MS: Multiple sclerosis
mTOR: mechanistic target of rapamycin
NLRP: nucleotide-binding oligomerization domain (NOD)
LRR: leucine-rich repeat
NLR: containing protein
PD: Parkinson's disease
RRMS: relapse-remission multiple sclerosis
SASP: senescence-associated secretory phenotype
TCR: T cell receptor
TCV: T cell vaccine
TGF-β: transforming growth factor-beta
Th1/2/17: T helper cell 1/2/17
TNFα: tumor necrosis factor alpha
Treg: regulatory T cell
WT: wild-type.
